# Rényi Entropy, Signed Probabilities, and the Qubit

**DOI:** 10.3390/e24101412

**Published:** 2022-10-03

**Authors:** Adam Brandenburger, Pierfrancesco La Mura, Stuart Zoble

**Affiliations:** 1 Stern School of Business, Tandon School of Engineering, NYU Shanghai, New York University, New York, NY 10012, USA; 2 HHL—Leipzig Graduate School of Management, 04109 Leipzig, Germany; 3 Signal Fox, Princeton, NJ 08542, USA

**Keywords:** Rényi entropy, signed probability, uncertainty principle, qubit

## Abstract

The states of the qubit, the basic unit of quantum information, are 2 × 2 positive semi-definite Hermitian matrices with trace 1. We contribute to the program to axiomatize quantum mechanics by characterizing these states in terms of an entropic uncertainty principle formulated on an eight-point phase space. We do this by employing Rényi entropy (a generalization of Shannon entropy) suitably defined for the signed phase-space probability distributions that arise in representing quantum states.

## 1. Introduction

The maximum entropy method was introduced into physics as a way of deriving the Boltzmann distribution of statistical mechanics (Jaynes [1]). In this paper, we apply entropy methods to characterize the basic unit of quantum information, namely, the qubit. Our work fits into the ongoing program to identify principles or axioms yielding quantum mechanics. This program goes back at least to Birkhoff and von Neumann [2] and their investigation of quantum mechanics as a non-classical logic. More recently, Hardy [3] reconstructed quantum theory from five axioms couched in terms of operations that can be conducted on a physical system. His work spurred many other axiomatizations based on communication complexity (Van Dam [4]), information causality (Pawlowski et al. [5]), information capacity (Dakić and Brukner [6]), and purification (Chiribella, et al. [7]), among other principles.

We aim to characterize the simplest quantum system, namely, a two-level system such as the spin of a particle. Empirically, the experimenter can observe a property such as spin in three arbitrarily chosen mutually orthogonal directions. In each direction, the outcome is binary (up or down). An empirical model gives the frequencies of these outcomes when identical copies of the same two-level system are prepared and one of the three measurements is performed on a given copy. We want to associate an entropy with an empirical model. This step is not immediate because entropy is a measure of the uncertainty in a single probability distribution, and an empirical model contains three distinct probability distributions (one for each direction). The solution is to move to phase space, where an empirical model is represented by a single probability distribution. Our phase space for a two-level system contains eight points, where each point specifies the outcome (up or down) of each of the three possible measurements. The possibility of a non-deterministic response to measurement—as in quantum mechanics—is allowed for by specifying a probability of each point in phase space. Later, we comment on the relationship between our phase-space framework and the finite-field representations due to Wootters and collaborators (e.g., Wootters [8]; Gibbons et al. [9]).

Our phase-space representation of an empirical model can be thought of as a particular (canonical) type of local hidden-variable model (Bell [10]), where the possible values of the hidden variable are precisely the possible points in phase space. It follows from Bell’s Theorem (Bell [10]) that there are empirical models which arise in quantum mechanics and which cannot be represented in phase space with ordinary probabilities. One answer is found in the Wigner distribution (Wigner [11]), which can take on negative values at certain locations in phase space. Dirac [12] and Feynman [13] also argued for admitting negative probabilities in quantum calculations. We emphasize that although we will allow phase-space probabilities to take negative values, the frequencies of all observable events remain non-negative. Further support for the use of negative probabilities comes from Abramsky and Brandenburger [14], who prove that the family of empirical models that can be represented in phase space this way is precisely the family of no-signaling theories (Popescu and Rohrlich [15]).

We are now ready to associate entropies with probability distributions on phase space. Within quantum mechanics, the most common entropy measure is the von Neumann entropy (von Neumann [16]). This is unsuitable for our purpose because it is defined within the quantum formalism, which we want to derive not assume. Shannon entropy (Shannon [17]) is also unsuitable when applied to probabilities in phase space, since, if the latter can be negative, then it would take complex values. Instead, we work with the more basic notion of Rényi entropy [18] and impose real-valuedness and smoothness. Rényi entropy satisfies the basic requirement of extensivity, i.e., it is additive across statistically independent systems. In fact, it is defined by this property together with some technical axioms (Daróczy [19]). Rényi entropy is used in various applications in quantum mechanics (Wehner and Winter [20]; Bialynicki-Birula and Rudnicki [21]; Coles et al. [22]).

The next step is to state an entropic uncertainty principle as an axiom on phase space. Note that different from other entropic uncertainty principles in quantum mechanics (Everett [23]; Hirschman [24]; Beckner [25]; Bialynicki-Birula and Mycielski [26]), our principle is formulated in phase space. Furthermore, we do not derive the principle from quantum mechanics but introduce it as an axiom. Our main result is that the set of probability distributions on phase space whose Rényi entropy exceeds a certain lower bound is exactly equal to the set of probability distributions that induce the qubit.

A paper that, broadly speaking, goes in the opposite direction to ours is Wootters and Sussman [27]. These authors work in a finite-field phase-space representation of discrete quantum systems and are able to identify certain minimum-uncertainty states. In particular, they show that a particular class of states (the “rotationally invariant states”) minimize Rényi 2-entropy (a special case of our family of entropy functionals, as we shall see).

We consider our axiomatization of the qubit as in line with the program enunciated by Fuchs [28] to find “deep *physical* principles” that yield quantum mechanics. This said, we do not claim that our axiom is self-evident. In relativity theory, the principle of light speed invariance is not an intuitive axiom—the point is that it is physically intelligible. (This comparison between quantum theory and relativity theory is also made in Onggadinata et al. [29].) Our interest in an uncertainty principle is similar. Ever since the initial formulation by Heisenberg [30], uncertainty principles have been viewed as one of the notably unintuitive features of quantum mechanics. However, even though they are mysterious at the everyday macroscopic level, uncertainty principles are physically interpretable, and they are evidently true of microscopic systems.

## 2. Preliminaries

A basis for the space of 2×2 Hermitian matrices is given by $\left\{\sigma_{0},\sigma_{1},\sigma_{2},\sigma_{3}\right\}$, where $\sigma_{0}=I$ is the 2×2 identity matrix and $\sigma_{1}$, $\sigma_{2}$, $\sigma_{3}$ are the Pauli matrices

σ1=0110,σ2=0−ii−0,σ3=1−00−1.

A 2×2 Hermitian matrix M satisfies Tr(M)=1 if and only if

$$M=\frac{1}{2}\left(I+r_{1}\sigma_{1}+r_{2}\sigma_{2}+r_{3}\sigma_{3}\right)$$

for some vector $r=\left(r_{1},r_{2},r_{3}\right)\in R^{3}$.

**Definition** **1.** *A 2×2 Hermitian matrix M with Tr(M)=1 is called a potential quantum state. If, in addition, M is positive semi-definite, then M is a quantum state, or a state of the qubit. We also refer to the corresponding vectors r as potential quantum states and quantum states.*

This is the model of the simplest quantum system, namely a two-level system such as the spin of a particle. Empirically, the experimenter can observe a property such as spin in three arbitrarily chosen mutually orthogonal directions $x_{1}$, $x_{2}$, and $x_{3}$. In each direction, the outcome of a measurement will be labeled +1 or −1. The expectation of the outcome in direction *i* is (see, e.g., p. 181 in Sakurai and Napolitano [31]).

$$\mathrm{Tr}\left(M\sigma_{i}\right)=r_{i}.$$

We want to associate an entropy with an empirical model. This step is not immediate because entropy is a measure of the uncertainty in a single probability distribution, and an empirical model contains three probability distributions (one for each direction). Our solution is to move to phase space, where an empirical model is represented by a single probability distribution. The *phase space* for a two-level system contains eight points,

$${\left\{+1,-1\right\}}^{3}=\left\{e_{n}\;|\;n=1,..,8\right\},$$

where $e_{n}\left(i\right)={\left(-1\right)}^{n_{i}}$ for $\left(n_{1},n_{2},n_{3}\right)$ the base-2 digits of n−1. Each point in phase space specifies the outcome of each of the three possible measurements. Non-deterministic responses to measurement are incorporated by specifying probabilities over the points in phase space. Let

$$Q=\{q\in R^{8}\;|\;\Sigma_{i=1}^{8}q_{i}=1\}$$

denote the set of all signed probability distributions on phase space. That is, we do not require the probabilities to be positive, only that they sum to 1. We define a map ϕ from *Q* to the set of potential quantum states by

$$\phi \left(q\right)=\frac{1}{2}\left(I+r_{1}\sigma_{1}+r_{2}\sigma_{2}+r_{3}\sigma_{3}\right),$$

where

$$r_{i}=\sum_{\left\{n|e_{n}\left(i\right)=+1\right\}}q_{n}\times \left(+1\right)\;+\sum_{\left\{n|e_{n}\left(i\right)=-1\right\}}q_{n}\times \left(-1\right).$$

The map ϕ gives the correct transformation from phase space to the space of potential quantum states, in the sense of preserving the empirical probabilities. This map is linear and it will be helpful to fix some notation surrounding a matrix representation. Note we have folded the condition that q is a probability distribution in as the last equation in the definition of representation below.

**Definition** **2.** *Let A denote the matrix*

1−1−1−1−1−1−1−11−1−1−1−1−1−1−11−1−1−1−1−1−1−11−1−1−1−1−1−1−1.

*For $r\in R^{3}$ define $\hat{r}=\left(r_{1},r_{2},r_{3},1\right)\in R^{4}$. For $q\in R^{8}$ and $r\in R^{3}$ we say q represents r if $Aq=\hat{r}$.*

## 3. Rényi Entropy

We are going to use phase space to formulate an entropic uncertainty principle as an axiom, and derive the quantum states this way. In particular, we will allow only those potential quantum states r for which there is a phase-space representation q whose entropy exceeds a lower bound. The non-classicality of the qubit becomes apparent because there are quantum states for which the only representations with entropy exceeding the bound are signed probability distributions. The use of negative probabilities on phase space to represent quantum systems goes back to the Wigner quasi-probability distribution (Wigner [11]). The first task then is to choose a suitable definition of entropy for signed probabilities.

We extend Rényi entropy ([18]) to signed probabilities and impose a smoothness condition that identifies a particular family of entropy functionals. Fix a finite set $X=\{x_{1},...,x_{n}\}$ together with an ordinary (unsigned) probability distribution q on *X*. Rényi entropy is the family of functionals

$$H_{\alpha }\left(q\right)=-\frac{1}{\alpha -1}\log_{2}\left(\sum_{i=1}^{n}\;q_{i}^{\alpha }\right),$$

where 0<α<∞ is a free parameter. (Shannon entropy is the special case, via L’Hôpital’s rule, when α=1.) We can preserve the real-valuedness of entropy under signed probabilities by taking absolute values

$$H_{\alpha }\left(q\right)=-\frac{1}{\alpha -1}\log_{2}(\sum_{i=1}^{n}\;|q_{i}{|}^{\alpha }).$$

This formula can also be derived axiomatically. (see Brandenburger and La Mura [32] who modify the original axioms for Rényi entropy in Rényi [18] and Daróczy [19].) We next impose a smoothness condition, requiring that $H_{\alpha }$ be smooth on the space of signed probabilities. That is, we require $H_{\alpha }\left(q_{1},\ldots ,q_{n-1},\left(1-\sum_{i=1}^{n-1}q_{i}\right)\right)$ to be $C^{\infty }$ on $R^{n-1}$. Now, if α is not an integer let *k* be the least integer with k>α. Then

$$\frac{\partial^{k}H_{\alpha }}{\partial q_{i}^{k}}\left(q\right)=\frac{f(q)}{g(q)},$$

where f(q)≠0 and g(q)=0 for any *q* with $q_{i}=0$. Thus, α must be an integer. If α is an odd integer then $\partial^{\alpha }H_{\alpha }/\partial q_{i}^{\alpha }\;\left(q\right)$ is undefined for *q* with $q_{i}=0$. Therefore, Rényi entropy takes the following form under our smoothness assumption.

**Definition** **3.** *Rényi entropy for signed probability distributions is the family of functionals*

$$H_{2k}\left(q\right)=-\frac{1}{2k-1}\log_{2}\left(\sum_{i=1}^{n}\;q_{i}{}^{2k}\right)=-\frac{2k}{2k-1}\log_{2}({∥q∥}_{2k}),$$

*where k=1,2,… is a free parameter.*

Finally in this section, we give an example of a quantum state such that the only representatives with Rényi entropy satisfying the lower bound are signed probabilities. Consider the quantum state $\left(r_{1},r_{2},r_{3}\right)=\left(\frac{1}{\surd 3},\frac{1}{\surd 3},\frac{1}{\surd 3}\right)$. Set k=1. The (unique) maximum 2-entropy representation is

$$q=\frac{1}{8}\left(1+\surd 3,1+\frac{1}{\surd 3},1+\frac{1}{\surd 3},1-\frac{1}{\surd 3},1+\frac{1}{\surd 3},1-\frac{1}{\surd 3},1-\frac{1}{\surd 3},1-\surd 3\right),$$

with negative final component. The 2-entropy of q is 2, which is the lower bound we impose below, so we cannot find a representation with all non-negative components with sufficiently high 2-entropy. In fact any state with $|r_{1}\left|+\right|r_{2}\left|+\right|r_{3}|>1$ will have this property.

## 4. Main Theorem

We can now state an entropic uncertainty principle as an axiom on phase space. The axiom is inspired by the use of entropic uncertainty relations in quantum information (Wehner and Winter [20]; Bialynicki-Birula and Rudnicki [21]; Coles et al. [22]).

**Uncertainty Principle**: A potential quantum state r satisfies the Uncertainty Principle if for every *k*, there is a phase-space probability distribution q that represents r and satisfies $H_{2k}\left(q\right)\geq 2$.

This says that we allow as potential quantum states only those states r containing a minimum amount of uncertainty, as measured by the entropy of a corresponding probability distribution q on phase space. Note that our Uncertainty Principle is a sequence of conditions, one for each *k*. This is because Rényi entropy itself is not a single functional but a sequence of functionals (indexed by *k*).

**Theorem** **1.** *The potential quantum states satisfying the Uncertainty Principle are precisely the states of the qubit.*

**Proof.**  We first show that the potential quantum states satisfying the Uncertainty Principle at k=1 are the states of the qubit. Note that

$$H_{2}\left(q\right)\geq 2\;\mathrm{if}\;\mathrm{and}\;\mathrm{only}\;\mathrm{if}\;{∥q∥}_{2}^{2}\leq \frac{1}{4}.$$

For a general r, the representation $q^{*}$ which maximizes 2-entropy is given by

$$q^{*}=A^{T}{\left(AA^{T}\right)}^{-1}\hat{r}.$$

Using the fact that $AA^{T}=8I$ we have

$$∥q^{*}{∥}_{2}^{2}={\hat{r}}^{T}{\left(AA^{T}\right)}^{-1}\hat{r}=\frac{1}{8}r^{T}r+\frac{1}{8}\leq \frac{1}{4}$$

if and only if

$$r_{1}^{2}+r_{2}^{2}+r_{3}^{2}\leq 1,$$

and the result follows since the matrix $\frac{1}{2}\left(I+r_{1}\sigma_{1}+r_{2}\sigma_{2}+r_{3}\sigma_{3}\right)$ is positive semi-definite if and only if $r_{1}^{2}+r_{2}^{2}+r_{3}^{2}\leq 1$.

We now show that if a potential state r satisfies the Uncertainty Principle at k=1 then it satisfies the Uncertainty Principle at all *k*. This is the main mathematical argument. Fix k>1 and let $r\in R^{3}$ be a state of the qubit. Choose a q to maximize the 2k-entropy of a representative of r. We want to show $H_{2k}\left(q\right)\geq 2$ which is equivalent to ${∥q∥}_{2k}\leq {\left(\frac{1}{2}\right)}^{\frac{2k-1}{k}}$.

Observe that q solves the norm minimization problem

$$\begin{array}{cc} \min_{\;q\in R^{8}}{∥q∥}_{2k}\\ \mathrm{subject}\mathrm{to}\; & Aq=\hat{r}. \end{array}$$

The dual problem is

$$\begin{array}{cc} \max_{\;x\in R^{4}}{\hat{r}}^{T}x\\ \mathrm{subject}\mathrm{to}\; & ∥A^{T}{x∥}_{\frac{2k}{2k-1}}\leq 1. \end{array}$$

(see pp. 221–222 in Boyd and Vandenberghe [33].) Note that ${∥\cdot ∥}_{\frac{2k}{2k-1}}$ is the dual norm of ${∥\cdot ∥}_{2k}$. Strong duality holds so the values of the primal and dual problems are equal. Let $y^{1},y^{k}$ be the maximizers of the dual problems for 2-entropy and 2k-entropy, respectively. Let

$$C_{1}=\{x\in R^{4}|∥A^{T}x{∥}_{2}\leq 1\}$$

and

$$C_{k}=\{x\in R^{4}|∥A^{T}x{∥}_{\frac{2k}{2k-1}}\leq 1\}.$$

Note that $C_{k}\subseteq C_{1}$ are both convex and, in fact, $C_{1}$ is the ball of radius $\frac{1}{\sqrt{8}}$. Let

$$z^{k}=({\hat{r}}^{T}y^{k}/∥\hat{r}{∥}_{2}^{2})\hat{r}$$

be the projection of $y^{k}$ onto $\hat{r}$. Since ${\hat{r}}^{T}y^{1}=\frac{∥\hat{r}{∥}_{2}}{\sqrt{8}}cos\;\theta$, where θ is the angle between them, we must have θ=0 and so

$$y^{1}=({\hat{r}}^{T}y^{1}/∥\hat{r}{∥}_{2}^{2})\hat{r}.$$

Since the values of the primal and dual problems are equal, these values are positive, so $\frac{∥z^{k}{∥}_{2}}{∥y^{1}{∥}_{2}}$ is equal to the ratio of the value of the general *k* problem to the value of the k=1 problem. By assumption

$${\hat{r}}^{T}y^{1}\leq \frac{1}{2},$$

so it is enough to show

$$\frac{∥z^{k}{∥}_{2}}{∥y^{1}{∥}_{2}}\leq {\left(\frac{1}{2}\right)}^{\frac{k-1}{k}}.$$

We will bound this expression by a function that can be explicitly maximized. Note that for every nonzero vector w there are unique λ<ν such that

$$∥A^{T}{\nu w∥}_{2}=1$$

and

$$∥A^{T}{\lambda w∥}_{\frac{2k}{2k-1}}=1.$$

This follows immediately from linearity, homogeneity, the fact that A has full rank, and the fact that $\frac{2k}{2k-1}<2$. Now let

$$f\left(w\right)=\frac{∥A^{T}{w∥}_{2}}{∥A^{T}{w∥}_{\frac{2k}{2k-1}}}.$$

**Figure 1:**
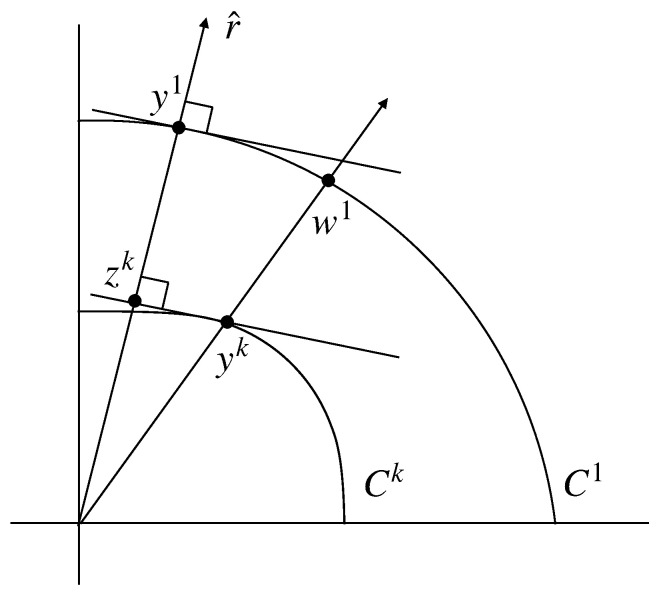
Comparing the maximizers of the dual problem.

By the previous observation and the fact that f(λw)=f(w) for any nonzero scalar λ, we see that f(w) is the ratio of the distance to the boundary of $C_{1}$ along the ray through w to the distance to the boundary of $C_{k}$. Let $w^{1}=\nu y^{k}$ belong to the boundary of $C_{1}$. Figure 1 depicts the situation in the plane containing $\hat{r}$ and $y^{k}$.

**Claim** **1.** $\frac{∥z^{k}{∥}_{2}}{∥y^{1}{∥}_{2}}$*is bounded by a value off*.

**Proof.**  We claim that

$$\frac{∥z^{k}{∥}_{2}}{∥y^{1}{∥}_{2}}\leq \frac{∥y^{k}{∥}_{2}}{∥w^{1}{∥}_{2}}.$$

Note that

$${\hat{r}}^{T}w^{1}\leq {\hat{r}}^{T}y^{1},$$

so the length of the projection of $w^{1}$ onto $\hat{r}$ (call this vector v) cannot exceed the length of $y^{1}$. By similar triangles then

$$\frac{∥y^{k}{∥}_{2}}{∥z^{k}{∥}_{2}}=\frac{∥w^{1}{∥}_{2}}{{∥v∥}_{2}}\geq \frac{∥w^{1}{∥}_{2}}{∥y^{1}{∥}_{2}},$$

so $\frac{∥z^{k}{∥}_{2}}{∥y^{1}{∥}_{2}}\leq \frac{∥y^{k}{∥}_{2}}{∥w^{1}{∥}_{2}}=f\left(w^{1}\right).$ □

To complete the proof of Theorem 1, it suffices to show that

$$\max\left\{f\left(w\right)|w\in R^{4}\right\}={\left(\frac{1}{2}\right)}^{\frac{k-1}{k}},$$

which we do in the Appendix A. □

## 5. Conclusions

We have shown that an entropic Uncertainty Principle formulated on an eight-point phase space characterizes the states of the qubit. We see our result as contributing to the program that aims to reconstruct quantum mechanics from physically interpretable axioms. Of course, our derivation is only for the simplest, two-level quantum system. We anticipate that to characterize an *n*-qubit system, methods will be needed that go beyond those in this paper. In particular, it may be necessary not only to extend our entropic Uncertainty Principle to the *n*-qubit case, but to identify new axioms. The Wootters and Sussman [27] analysis may be an important guide in this direction in that they are able to identify certain minimum-uncertainty states in an *n*-qubit system. A full characterization may be possible combining techniques across the two papers.

We re-emphasize that our paper is aimed at a derivation not a representation of the qubit. This explains why our phase space contains eight points while in Wootters [8] and Gibbons et al. [9] the phase space for a single qubit comprises four points. A four-point space is the appropriate domain for the discrete Wigner function, but we do not assume a Wigner representation.

A related derivation of the qubit is Onggadinata et al. [29]. Similar to our paper, they employ Rényi entropy, but fix α=2. This instance of Rényi entropy is often called collision entropy. Their postulate is that the collision entropy is constant under any dynamics (not necessarily deterministic) on a finite one-dimensional lattice. From this, they recover the qubit with its full dynamics as defined on the Bloch sphere.

Both Wootters and Sussman [27] and Onggadinata et al. [29] work with Rényi 2-entropy. By contrast, we are able to obtain our results not just for 2-entropy, but for the entire family of 2k-entropies, which we derived from basic principles. It would be interesting to see if our methods could be employed to generalize the results in these papers.

## Data Availability

No new data were created or analyzed in this study. Data sharing is not applicable to this article.

## Appendix A

Let $w\in R^{4}$ and v=wA. Let $t\in R^{8}$ be defined by $t_{i}=v_{i}^{\frac{1}{2k-1}}$. Note that the critical points of *f* are the same as the critical points of

$$\frac{f^{2}\left(w\right)}{8}=\frac{{∥v∥}_{2}^{2}}{{8∥v∥}_{2k/2k-1}^{2}}=\frac{wAA^{T}w^{T}}{{8∥v∥}_{2k/2k-1}^{2}}=\frac{{∥w∥}_{2}^{2}}{{∥v∥}_{2k/2k-1}^{2}},$$

which are the solutions of the system of first-order conditions

$$w_{i}=h\left(w\right)r_{i}t^{T}\;\;i=1,2,3,4,$$

where

$$h\left(w\right)=\frac{{∥w∥}_{2}^{2}}{{∥v∥}_{2k/2k-1}^{2k/2k-1}}>0$$

and $r_{i}$ is the *i*th row of the matrix A. It is helpful to write out the system with γ denoting $\frac{1}{2k-1}$ for readability:

$$w_{1}=h\left(w\right)[{\left(w_{1}+w_{2}+w_{3}+w_{4}\right)}^{\gamma }-{\left(-w_{1}+w_{2}+w_{3}+w_{4}\right)}^{\gamma }+{\left(w_{1}-w_{2}+w_{3}+w_{4}\right)}^{\gamma }-$$

$${\left(-w_{1}-w_{2}+w_{3}+w_{4}\right)}^{\gamma }+{\left(w_{1}+w_{2}-w_{3}+w_{4}\right)}^{\gamma }-{\left(-w_{1}+w_{2}-w_{3}+w_{4}\right)}^{\gamma }+$$

$${\left(w_{1}-w_{2}-w_{3}+w_{4}\right)}^{\gamma }-{\left(-w_{1}-w_{2}-w_{3}+w_{4}\right)}^{\gamma }],$$

$$w_{2}=h\left(w\right)[{\left(w_{1}+w_{2}+w_{3}+w_{4}\right)}^{\gamma }+{\left(-w_{1}+w_{2}+w_{3}+w_{4}\right)}^{\gamma }-{\left(w_{1}-w_{2}+w_{3}+w_{4}\right)}^{\gamma }-$$

$${\left(-w_{1}-w_{2}+w_{3}+w_{4}\right)}^{\gamma }+{\left(w_{1}+w_{2}-w_{3}+w_{4}\right)}^{\gamma }+{\left(-w_{1}+w_{2}-w_{3}+w_{4}\right)}^{\gamma }-$$

$${\left(w_{1}-w_{2}-w_{3}+w_{4}\right)}^{\gamma }-{\left(-w_{1}-w_{2}-w_{3}+w_{4}\right)}^{\gamma }],$$

$$w_{3}=h\left(w\right)[{\left(w_{1}+w_{2}+w_{3}+w_{4}\right)}^{\gamma }+{\left(-w_{1}+w_{2}+w_{3}+w_{4}\right)}^{\gamma }+{\left(w_{1}-w_{2}+w_{3}+w_{4}\right)}^{\gamma }+$$

$${\left(-w_{1}-w_{2}+w_{3}+w_{4}\right)}^{\gamma }-{\left(w_{1}+w_{2}-w_{3}+w_{4}\right)}^{\gamma }-{\left(-w_{1}+w_{2}-w_{3}+w_{4}\right)}^{\gamma }-$$

$${\left(w_{1}-w_{2}-w_{3}+w_{4}\right)}^{\gamma }-{\left(-w_{1}-w_{2}-w_{3}+w_{4}\right)}^{\gamma }],$$

$$w_{4}=h\left(w\right)[{\left(w_{1}+w_{2}+w_{3}+w_{4}\right)}^{\gamma }+{\left(-w_{1}+w_{2}+w_{3}+w_{4}\right)}^{\gamma }+{\left(w_{1}-w_{2}+w_{3}+w_{4}\right)}^{\gamma }+$$

$${\left(-w_{1}-w_{2}+w_{3}+w_{4}\right)}^{\gamma }+{\left(w_{1}+w_{2}-w_{3}+w_{4}\right)}^{\gamma }+{\left(-w_{1}+w_{2}-w_{3}+w_{4}\right)}^{\gamma }+$$

$${\left(w_{1}-w_{2}-w_{3}+w_{4}\right)}^{\gamma }+{\left(-w_{1}-w_{2}-w_{3}+w_{4}\right)}^{\gamma }].$$

**Claim** **A1.**
*The system* $w=h\left(w\right)At^{T}$ *has the following properties:*

1. *If*w*is a solution then so is*λw*for any*λ≠0.
2. *If*w*is a solution then*v*is a solution, where*v*is obtained from*w*by permuting coordinates*.

**Proof.** For (1) we have $h\left(\lambda w\right)t^{T}\left(\lambda w\right)=\frac{\lambda^{2}\lambda^{1/2k-1}}{\lambda^{2k/2k-1}}h\left(w\right)t^{T}=\lambda w$. For (2) we have

$$w_{1}=h\left(w_{4},w_{2},w_{3},w_{1}\right)r_{4}t^{T}\left(w_{4},w_{2},w_{3},w_{1}\right)$$

and

$$w_{4}=h\left(w_{4},w_{2},w_{3},w_{1}\right)r_{1}t^{T}\left(w_{4},w_{2},w_{3},w_{1}\right),$$

and similarly for $w_{2},w_{3}$. □

**Claim** **A2.**
*Assume* $w_{4}\neq 0$ *. Let* i,j<4 *. Then*

$$|w_{i}\left|=\right|w_{j}|\;or\;w_{i}w_{j}=0.$$

**Proof.** We may assume $w_{4}>0$. For *N* sufficiently large we have ${∥w-a∥}_{2}<{∥a∥}_{2}$ where a=(0,0,0,N). Thus, the Taylor series expansion of $r_{i}t^{T}$ at the point a converges at w. We have

$$w_{1}=8h\left(w\right)\sum_{\begin{array}{c} \alpha_{1}\in O\\ \alpha_{2},\alpha_{3}\in E\\ \alpha_{4}\in N \end{array}}\frac{{\left(w-a\right)}^{\alpha }}{\alpha !}C\left(\sum_{i=1}^{4}\alpha_{i}\right),$$

$$w_{2}=8h\left(w\right)\sum_{\begin{array}{c} \alpha_{2}\in O\\ \alpha_{1},\alpha_{3}\in E\\ \alpha_{4}\in N \end{array}}\frac{{\left(w-a\right)}^{\alpha }}{\alpha !}C\left(\sum_{i=1}^{4}\alpha_{i}\right),$$

$$w_{3}=8h\left(w\right)\sum_{\begin{array}{c} \alpha_{3}\in O\\ \alpha_{1},\alpha_{2}\in E\\ \alpha_{4}\in N \end{array}}\frac{{\left(w-a\right)}^{\alpha }}{\alpha !}C\left(\sum_{i=1}^{4}\alpha_{i}\right),$$

$$w_{4}=8h\left(w\right)\sum_{\begin{array}{c} \alpha_{1},\alpha_{2},\alpha_{3}\in E\\ \alpha_{4}\in N \end{array}}\frac{{\left(w-a\right)}^{\alpha }}{\alpha !}C\left(\sum_{i=1}^{4}\alpha_{i}\right),$$

where $\alpha \in N^{4}$ is a multi-index, N=E∪O={0,2,4,...}∪{1,3,5,...},

$$\alpha !=\alpha_{1}!\alpha_{2}!\alpha_{3}!\alpha_{4}!,$$

$${\left(w-a\right)}^{\alpha }=w_{1}^{\alpha_{1}}w_{2}^{\alpha_{2}}w_{3}^{\alpha_{3}}{\left(w_{4}-N\right)}^{\alpha_{4}},$$

and *C* is defined by

$$C\left(0\right)=1,\;C\left(1\right)=\frac{1}{\left(2k-1\right)N^{\frac{2k}{2k-1}}},\;\mathrm{and}$$

$$C\left(n\right)=\frac{{\left(-1\right)}^{n-1}\prod_{j=1}^{n-1}\left(j\left(2k-1\right)-1\right)}{{\left(2k-1\right)}^{n}N^{\frac{n(2k-1)-1}{2k-1}}}\;for\;n>1.$$

Note that $C(\sum_{i=1}^{4}\alpha_{i})>0$ if and only if $\sum_{i=1}^{4}\alpha_{i}\in O$. Assume $w_{1},w_{2}\neq 0$. We have

$$w_{1}\sum_{\begin{array}{c} \alpha_{2}\in O\\ \alpha_{1},\alpha_{3}\in E\\ \alpha_{4}\in N \end{array}}\frac{{\left(w-a\right)}^{\alpha }}{\alpha !}C\left(\sum_{i=1}^{4}\alpha_{i}\right)=w_{2}\sum_{\begin{array}{c} \alpha_{1}\in O\\ \alpha_{2},\alpha_{3}\in E\\ \alpha_{4}\in N \end{array}}\frac{{\left(w-a\right)}^{\alpha }}{\alpha !}C\left(\sum_{i=1}^{4}\alpha_{i}\right),$$

equivalently

$$\sum_{\begin{array}{c} \alpha_{2}\in O\\ \alpha_{1},\alpha_{3}\in E\\ \alpha_{4}\in N \end{array}}\frac{{\left(w-a\right)}^{\alpha +(1,0,0,0)}}{\alpha !}C\left(\sum_{i=1}^{4}\alpha_{i}\right)=\sum_{\begin{array}{c} \alpha_{1}\in O\\ \alpha_{2},\alpha_{3}\in E\\ \alpha_{4}\in N \end{array}}\frac{{\left(w-a\right)}^{\alpha +(0,1,0,0)}}{\alpha !}C\left(\sum_{i=1}^{4}\alpha_{i}\right).$$

Re-indexing we have

$$\sum_{\begin{array}{c} \alpha_{1},\alpha_{2}\in O\\ \alpha_{3}\in E\\ \alpha_{4}\in N \end{array}}\alpha_{1}\frac{{\left(w-a\right)}^{\alpha }}{\alpha !}C\left(\sum_{i=1}^{4}\alpha_{i}-1\right)=\sum_{\begin{array}{c} \alpha_{1},\alpha_{2}\in O\\ \alpha_{3}\in E\\ \alpha_{4}\in N \end{array}}\alpha_{2}\frac{{\left(w-a\right)}^{\alpha }}{\alpha !}C\left(\sum_{i=1}^{4}\alpha_{i}-1\right).$$

Collecting terms we have

$$\sum_{\begin{array}{c} \alpha_{1}<\alpha_{2}\\ \alpha_{1},\alpha_{2}\in O\\ \alpha_{3}\in E\\ \alpha_{4}\in N \end{array}}\left(\alpha_{1}-\alpha_{2}\right)\left(w_{1}^{\alpha_{1}}w_{2}^{\alpha_{2}}-w_{1}^{\alpha_{2}}w_{2}^{\alpha_{1}}\right)w_{3}^{\alpha_{3}}{\left(w_{4}-N\right)}^{\alpha_{4}}\frac{C(\sum_{i=1}^{4}\alpha_{i}-1)}{\alpha !}=0,$$

equivalently

$$\sum_{\begin{array}{c} \alpha_{1}<\alpha_{2}\\ \alpha_{1},\alpha_{2}\in O\\ \alpha_{3}\in E\\ \alpha_{4}\in N \end{array}}\left(\alpha_{1}-\alpha_{2}\right)w_{1}^{\alpha_{1}}w_{2}^{\alpha_{2}}\left(1-{\left(\frac{w_{1}}{w_{2}}\right)}^{\alpha_{2}-\alpha_{1}}\right)w_{3}^{\alpha_{3}}{\left(w_{4}-N\right)}^{\alpha_{4}}\frac{C(\sum_{i=1}^{4}\alpha_{i}-1)}{\alpha !}=0.$$

The key point is that

$$\left(\alpha_{1}-\alpha_{2}\right)w_{1}^{\alpha_{1}}w_{2}^{\alpha_{2}}w_{3}^{\alpha_{3}}{\left(w_{4}-N\right)}^{\alpha_{4}}\frac{C(\sum_{i=1}^{4}\alpha_{i}-1)}{\alpha !}$$

always has the same sign as $w_{1}w_{2}$. Thus, since $\alpha_{2}-\alpha_{1}\in E$ we conclude that $|w_{1}\left|=\right|w_{2}|$. □

**Claim** **A3.** *If*$w_{i},w_{j}\neq 0$*then*$|w_{i}\left|=\right|w_{j}|$.

**Proof.** By Claim A1 and Claim A2 we may assume that $w_{1},w_{4}\neq 0$ and $w_{2},w_{3}=0$. We may further assume that $w_{4}=1$. Thus, the equation for $w_{1}$ becomes

$$w_{1}=\frac{{\left(w_{1}+1\right)}^{1/2k-1}-{\left(-w_{1}+1\right)}^{1/2k-1}}{{\left(w_{1}+1\right)}^{1/2k-1}+{\left(-w_{1}+1\right)}^{1/2k-1}}$$

so

$$\left(w_{1}+1\right){\left(-w_{1}+1\right)}^{1/2k-1}=\left(-w_{1}+1\right){\left(w_{1}+1\right)}^{1/2k-1},$$

from which we conclude that $w_{1}\in \left\{-1,1\right\}$ as desired. □

We have thus shown that for every i,j≤4 either $|w_{i}\left|=\right|w_{j}|$ or $w_{i}w_{j}=0$ so we need only consider critical points with

$$w_{i}\in \left\{0,1,-1\right\}$$

for each i=1,...,4. It is easy to check that the maximum of the original function *f* occurs when exactly two of the weights are 0 and this maximum value is ${\left(\frac{1}{2}\right)}^{\frac{k-1}{k}}$, completing the proof of Theorem 1.
